# S100A8/A9 and S100A12 Proteins and Macrophage Polarization: Therapeutic Targets in Atherosclerosis

**DOI:** 10.3390/biom16081115

**Published:** 2026-07-30

**Authors:** Atreya J. Kulkarni, Vikrant Rai

**Affiliations:** 1 River Islands High School, 16601 Riptide Wy, Lathrop, CA 95330, USA; atreyakul@gmail.com; 2 Department of Translational Research, Western University of Health Sciences, 309 E. Second Street, Pomona, CA 91766, USA

**Keywords:** atherosclerosis, S100A8/A9, S100A12, macrophage polarization, inflammation, plaque vulnerability

## Abstract

Atherosclerosis is a chronic inflammatory disease characterized by the accumulation of lipids, immune cells, and fibrotic tissue within the arterial wall. Among the immune cells that drive this process, macrophages play a central role by mediating both inflammatory activation and tissue repair. Their polarization into either pro-inflammatory (M1) or anti-inflammatory (M2) phenotypes determines whether plaque progression or stabilization occurs. S100 proteins, particularly S100A8, S100A9, and S100A12, are critical regulators of macrophage function in atherosclerosis. Acting as damage-associated molecular patterns, these calcium-binding proteins interact with receptors such as receptor for advanced glycation end products (RAGE) and toll-like receptor (TLR)-4 to sustain inflammatory signaling, promote oxidative stress, and amplify cytokine production within atherosclerotic plaques. Elevated S100 protein levels correlate with increased macrophage infiltration, plaque instability, and heightened cardiovascular risk. Understanding how S100 proteins influence macrophage polarization offers new insights into the mechanisms underlying chronic vascular inflammation. Targeting the S100A8/A9 and S100A12 pathways represents a promising therapeutic strategy to mitigate macrophage-driven inflammation and improve plaque stability. Approaches such as inhibition of S100 protein-receptor interactions, suppression of downstream reactive oxygen species production, and modulation of macrophage polarization toward the M2 phenotype have shown potential in experimental models. This narrative review explores the roles of macrophages in atherosclerosis, the involvement of S100 proteins in both disease progression and macrophage polarization, and the therapeutic implications of targeting S100 protein–mediated immune responses. Together, these findings highlight S100 proteins as a therapeutic target modulating macrophage polarization to attenuate atherosclerotic plaque vulnerability.

## 1. Introduction

**Figure 1:**
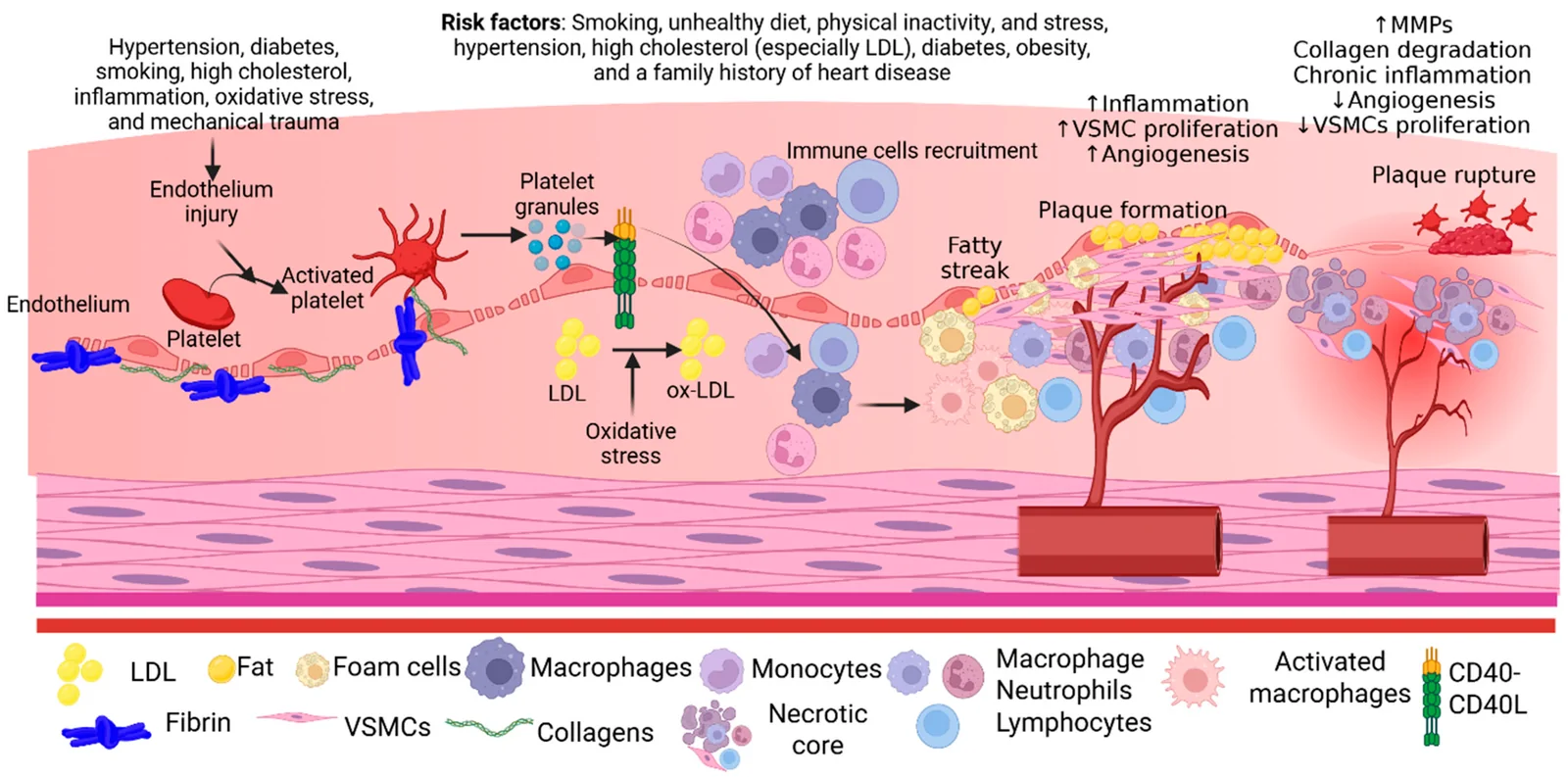
Molecular and cellular mechanisms involved in the pathogenesis of plaque formation and rupture. Atherosclerotic plaque formation and rupture involve molecular mechanisms driven by endothelial dysfunction, lipid accumulation, and chronic inflammation. Key processes include ow-density lipoprotein (LDL) oxidation, foam cell formation, and matrix metalloproteinases (MMPs) degrading fibrous cap. ↑—increased and ↓—decreased. Created in BioRender. Rai, V. (2026) https://BioRender.com/zyc08p2.

Atherosclerosis is a progressive cardiovascular disease characterized by lipid accumulation, chronic inflammation, and structural remodeling of the arterial wall [1,2]. The disease begins with endothelial activation, which is triggered by risk factors such as hyperlipidemia, hypertension, or oxidative stress [1,3]. This activation promotes the infiltration of circulating monocytes into the arterial intima, where they differentiate into macrophages and initiate local inflammation [4,5]. Over time, lipid retention, immune cell infiltration, and expansion of the necrotic core destabilize plaques, increasing the likelihood of rupture and thrombosis [4,6]. Plaque rupture is the underlying cause of acute clinical events such as myocardial infarction and ischemic stroke, highlighting the importance of understanding the mechanisms that govern plaque development and vulnerability [1,2] (Figure 1).

Among the immune cells involved in atherosclerosis, including macrophages, T cells, B cells, neutrophils, dendritic cells, and natural killer (NK) cells, macrophages play a central role in both driving the progression and facilitating the resolution [3,4]. They contribute to foam cell formation, secrete pro-inflammatory cytokines, and regulate efferocytosis, the clearance of apoptotic cells that is essential for plaque stability [4,7]. Recent work has expanded the view of macrophages beyond the traditional dichotomy of pro-inflammatory M1 and anti-inflammatory M2 phenotypes, instead identifying a spectrum of macrophage states within plaques, each shaped by distinct microenvironmental cues [8,9]. The plasticity of these macrophages allows them to either amplify vascular inflammation or promote tissue repair, making them a critical determinant of plaque fate [10,11].

S100 calcium-binding proteins, particularly S100A8, S100A9, and S100A12, have emerged as important regulators of vascular inflammation and macrophage biology [12,13,14]. These proteins, released from immune cells such as monocytes, neutrophils (granulocytes), macrophages, dendritic cells, and Langerhans cells during stress or injury, act as damage-associated molecular patterns (DAMPs) that signal through receptors such as receptors for advanced glycation end products (RAGE) and Toll-like receptors (TLRs), mainly TLR4 [15,16]. Their expression correlates with cardiovascular disease severity, and accumulating evidence suggests that they directly influence macrophage recruitment, activation, and polarization [17,18,19]. As macrophage polarization affects plaque stability, studying how S100 proteins guide macrophage phenotypes offer a promising strategy for developing targeted therapies [20,21]. We have focused on S100A8/A9 and S100A12 because of their significantly increased expression in atherosclerotic plaques [13,22,23]. This review focuses on summarizing the role of macrophages, inflammation, and S100 proteins in atherosclerosis, followed by connecting the links to elucidate the role of S100 proteins (S100A8, S100A9, and S100A12) in macrophage polarization. Finally, the review will delve into considering how targeting S100-mediated macrophage signaling may reduce plaque vulnerability.

## 2. Macrophages and Atherosclerosis

**Figure 2:**
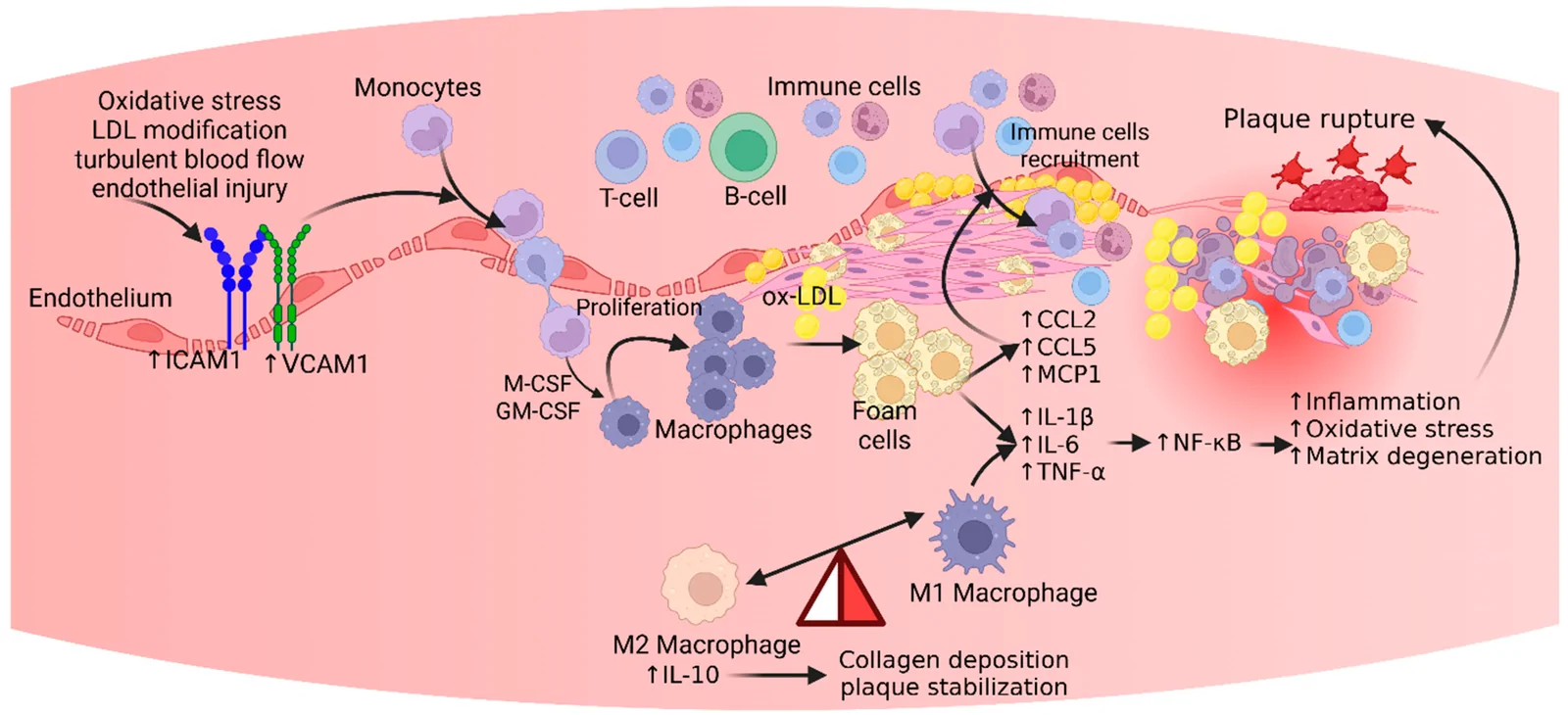
Role of macrophages in plaque formation and vulnerability. Macrophages play a critical role in plaque formation via the formation of foam cells, contributing to inflammation and collagen degradation. An imbalance between proinflammatory (M1) and anti-inflammatory (M2) macrophages contributes to the progression of plaque and vulnerability. Macrophage colony-stimulating factor (M-CSF) and granulocyte-macrophage colony-stimulating factor (GM-CSF), interleukin (IL), tumor necrosis factor (TNF)-α, nuclear factor kappa beta (NF-κB), intercellular adhesion molecule 1 (ICAM1), vascular cell adhesion molecule 1 (VCAM-1), chemokine (C-C motif) ligand 2 (CCL2), chemokine (C-C motif) ligand 5 (CCL5), monocyte chemoattractant protein 1 (MCP1), minimally oxidized low-density lipoprotein (ox-LDL). ↑—increased and ↓—decreased. Created in BioRender. Rai, V. (2026) https://BioRender.com/qj7q3ct.

Macrophages are among the first immune cells recruited to develop atherosclerotic lesions and remain one of the most abundant immune cell populations in advanced plaques [4,5]. Their recruitment is initiated when endothelial cells, activated by oxidized low-density lipoproteins (ox-LDL) and disturbed blood flow, upregulate adhesion molecules such as vascular cell adhesion protein 1 (VCAM-1) and intercellular adhesion molecule 1 (ICAM-1) and secrete chemokines like C-C motif chemokine ligand 2 (CCL2) [1,3]. Circulating monocytes adhere to the endothelium, migrate into the intima, and differentiate into macrophages under the influence of growth factors, including macrophage colony-stimulating factor (M-CSF) and granulocyte-macrophage colony-stimulating factor (GM-CSF) [4,24]. In addition to monocyte-derived infiltration, macrophages also expand locally within plaque through in situ proliferation, further contributing to their accumulation [25] (Figure 2).

Once in the intima, macrophages ingest minimally oxidized LDL through scavenger receptors such as CD36 and SR-A [4,26]. This unregulated lipid uptake leads to the formation of foam cells, a hallmark of early atherosclerotic lesions [4,5]. Foam cells initially serve a protective role by sequestering lipids, but their continued accumulation eventually causes cellular dysfunction [4]. Foam cells release pro-inflammatory cytokines such as interleukin (IL)-1, IL-6, and tumor necrosis factor-alpha (TNF-α), along with chemokines like CCL2 (also known as monocyte chemoattractant protein 1 (MCP-1)) and C-C motif chemokine ligand 5 (CCL5) (Figure 2), undergo apoptosis, and lose their ability to perform efferocytosis efficiently [4,7]. Defective efferocytosis allows apoptotic debris to accumulate, forming the necrotic core that destabilizes the plaque [6,7].

The functional diversity of macrophages in atherosclerosis extends beyond the classical M1/M2 polarization model [11,27]. Pro-inflammatory macrophages (M1) secrete cytokines such as TNF-α, IL-1β, and IL-6 and contribute to oxidative stress and matrix degradation, processes that weaken the fibrous cap and increase the risk of rupture [2,3]. Conversely, reparative macrophages (M2) secrete anti-inflammatory mediators like IL-10, promote collagen deposition, and support plaque stabilization [24,27] (Figure 2). Recent transcriptomic studies have identified specialized macrophage subsets, including TREM2^high^ foam cell–like macrophages, oxidative stress–induced Mox macrophages, and heme-handling macrophages such as Mhem and M4, underscoring the complexity of macrophage biology within plaques [8,28]. In the context of murine atherosclerosis, Mhem macrophages are atheroprotective and resistant to foam cell formation, Mox macrophages (induced by oxidized phospholipids) are proatherogenic, and M4 macrophages (polarized by platelet factor 2) are atherogenic because they increase secretion of IL-6 and TNF-α and show higher expression of matrix metalloproteinase (MMP)-7 and S100A8 [29]. Triggering receptor expressed on myeloid cells (TREM)2^high^ foam cell–like macrophages drive foam cell formation and lesion growth. However, in advanced disease, TREM2 protects by promoting macrophage survival and efferocytosis (the clearing of dead cells) to limit necrotic core formation and stabilize plaques [30]. The studies related to these four subtypes of macrophages in relation to atherosclerosis and in humans are scarce and should be investigated. Further, other than increased expression of S100A8 on TREM2^high^ macrophages, the relation of S100A8/A9 and S100A12 with these subtypes of macrophages in the context of atherosclerosis remains poorly understood. Tissue-resident macrophages (TRMs) and resident-like macrophages (res-like) maintain arterial wall homeostasis, but in atherosclerosis, they are overwhelmed by lipid accumulation. Located primarily in the vascular adventitia, these embryo-derived cells help preserve tissue integrity until recruited monocyte-derived macrophages trigger chronic inflammation, form foam cells, and drive plaque growth [31]. Instead of maintaining a homeostatic state, resident-like macrophages exposed to S100 proteins can shift toward mixed activation phenotypes or undergo metabolic reprogramming that fuels chronic inflammation. Once activated, these macrophages are stimulated to produce even more S100 proteins [32,33]. In established atherosclerotic plaques, local proliferation of macrophages dominates plaque growth, outpacing the recruitment of circulating blood monocytes. This local division drives the expansion of lipid-laden foam cells, forming a necrotic core that can destabilize the artery wall and trigger cardiovascular events [10,34]. Rapid local proliferation of macrophages is called macrophage activation syndrome [35] and massive systemic release of S100A8/A9 and S100A12 acts as a driving force behind this severe inflammatory cascade involving macrophage differentiation, pro-inflammatory cytokines, and TLR-4 and RAGE activation [13]. The critical role of macrophages in atherosclerotic plaques is supported by the findings of selectively upregulated functional pathways, including antigen presentation, hypoxic response, inflammation, lipid metabolism, cell proliferation, apoptosis, and cellular energetics in symptomatic plaque’s macrophages involving IL-1β, TNF-α, and nuclear factor kappa beta (NF-κB) [36].

Macrophage polarization is the process by which macrophages shift between M1 and M2 states, crucial for controlling inflammation: M1 macrophages initiate inflammation to fight pathogens, releasing cytokines like TNF-α and IL-1β; and M2 macrophages resolve it, promote tissue repair, and release anti-inflammatory mediators like IL-10. Homeostasis relies on switching between these roles, but imbalances can lead to chronic inflammatory diseases, making M1/M2 regulation a therapeutic target [37,38]. Macrophage polarization is crucial in atherosclerosis, shifting macrophages between M1 and M2 states, which dictates plaque progression, stability, and rupture. But the balance is often disrupted in disease, with therapies targeting this balance showing promise for cardiovascular health. Macrophages exhibit high heterogeneity and plasticity in atherosclerotic plaques. This contributes to the dynamic progression of atherosclerosis, plaque formation, and transition into vulnerable plaques and plaque vulnerability [10]. Ultimately, macrophages dictate whether a lesion progresses toward vulnerability or stabilizes [4,5] (Figure 2). Their secretory products influence smooth muscle cell behavior, extracellular matrix (ECM) remodeling, and vascular calcification, while their survival or death shapes the composition of the necrotic core [3,6]. Dysregulation of macrophage function, particularly impaired efferocytosis and persistent pro-inflammatory signaling, is a defining feature of unstable plaques [2,7]. Thus, understanding the molecular pathways that govern macrophage proliferation and migration, including those modulated by S100A8/A9 and S100A12 proteins, is essential for identifying new therapeutic strategies to prevent atherosclerotic complications [13,21,39]. The concept of involving S100 proteins (S100A8/A9 and S100A12) is important because these proteins regulate macrophage-mediated inflammation [13]. The notion of targeting S100A8/A9 and S100A12 proteins associated with macrophage inflammation is supported by the findings of attenuated LPS-induced acute lung injury involving TLR4/MyD88/NF-κB signaling pathway via S100A9 gene deficiency regulating M1 macrophage polarization [40]. This suggests the probable role of S100A8/A9 and S100A12 in macrophage polarization in atherosclerosis, which remains unexplored and warrants future research.

## 3. Inflammation and Atherosclerosis

Chronic vascular inflammation is the central force that drives every stage of atherosclerosis, from the earliest endothelial changes to plaque rupture [2,3]. Macrophages sit at the heart of this process [4,5]. Their recruitment, activation, and secretory behavior shape the inflammatory landscape inside the arterial wall and determine whether a lesion grows, stabilizes, or destabilizes [1,4]. Once monocytes enter the intima and differentiate into macrophages, they are exposed to modified lipoproteins, oxidative stress, and cytokines that shift them toward a persistently activated state [27,38]. This activation leads to the continuous release of pro-inflammatory cytokines such as IL-1β, IL-6, TNF-α, and chemokines that attract more monocytes, creating a self-sustaining inflammatory loop [2,4] (Figure 1 and Figure 2).

**Figure 3:**
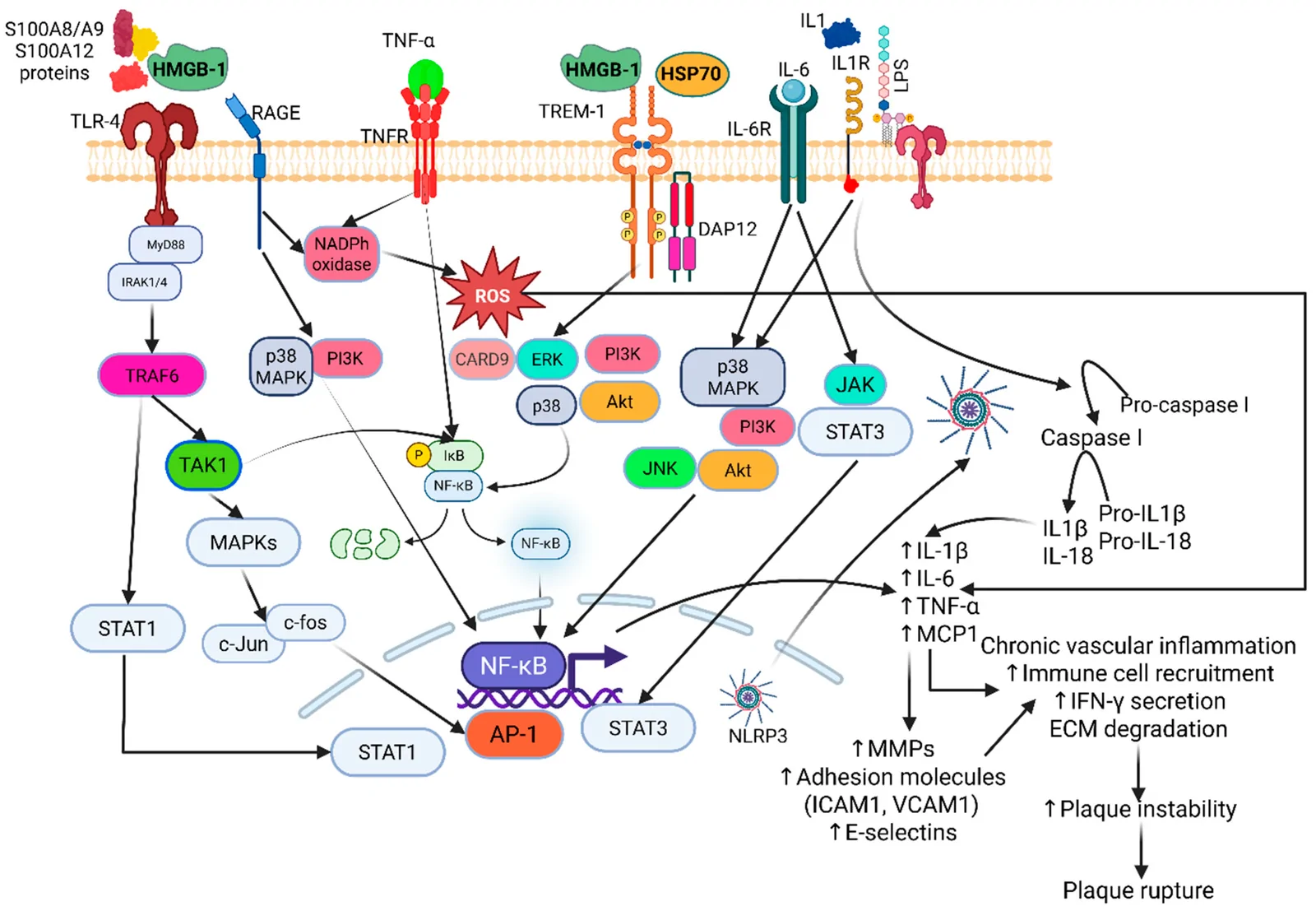
Inflammation promotes plaque vulnerability. Chronic vascular inflammation actively drives atherosclerosis progression and promotes stable plaques into vulnerable plaques prone to rupture. This is mediated by S100 proteins (mainly S100A8/A9 and S100A12), NOD-, LRR- and pyrin domain-containing protein 3 (NLRP3) inflammasomes, macrophages, toll-like receptors (TLRs), and pro-inflammatory cytokines and associated inflammatory pathways. High mobility group box protein (HMGB)-1, triggering receptor expressed on myeloid cells (TREM)-1, DNAX activating protein of 12 kDa (DAP12), tumor necrosis factor receptor (TNFR), tumor necrosis factor (TNF)-α, interleukin (IL), lipopolysaccharide (LPS), myeloid differentiation primary response 88 (MYD88), Interleukin-1 receptor-associated kinases 1 and 4 (IRAK1/4), tumor Necrosis Factor Receptor-Associated Factor 6 (TRAF6), extracellular signal-regulated kinase (ERK), phosphoinositide 3-kinase (PI3K), protein kinase B (Akt), nuclear factor kappa beta (NF-κB), extracellular matrix (ECM), signal transducer and activator of transcription (STAT). ↑—increased and ↓—decreased. Created in BioRender. Rai, V. (2026) https://BioRender.com/6u60hiv.

One of the most important mechanisms linking macrophages to vascular inflammation is activation of the NLR family pyrin domain containing 3 (NLRP3) inflammasome [41,42]. The NLRP3 inflammasome is a cytosolic protein complex that senses sterile danger signals commonly found in atherosclerotic lesions, including cholesterol crystals, oxidized LDL, and reactive oxygen species (ROS) [41,43]. When activated, NLRP3 triggers cleavage of pro-IL-1β and pro-IL-18 into their active forms through caspase-1, amplifying the inflammatory response [42,43]. IL-1β, in particular, has potent effects within plaques. It increases endothelial adhesion molecule expression, stimulates smooth muscle cell activation, and further enhances macrophage recruitment and cytokine production [2,3]. This creates a cycle where inflammation promotes plaque progression, and plaque progression generates more inflammatory stimuli [4,5] (Figure 3).

Macrophage lipid handling also contributes to inflammasome activation. As macrophages take up oxidized or aggregated LDL, they accumulate cholesterol crystals [4,41]. These crystals destabilize lysosomes, a key upstream trigger for NLRP3 activation [41,42]. This ties foam cell formation directly to inflammasome signaling [2,4]. Over time, sustained NLRP3 activation leads to increased pyroptosis, an inflammatory form of cell death [44]. Pyroptotic macrophages release intracellular contents, including additional inflammatory cues and unprocessed lipids, which feed the necrotic core and weaken the fibrous cap [6,44].

Inflammasome signaling does not act in isolation. It interacts with pathways already active in atherosclerosis, such as TLR4 and RAGE signaling triggered by S100 proteins [15,45] (Figure 3). These inputs work together to maintain macrophages in an M1-like inflammatory state [13,27]. In turn, IL-1β and IL-18 push the plaque microenvironment toward sustained inflammation, making it harder for reparative or M2-like responses to take hold [10,11]. The result is impaired efferocytosis, greater foam cell death, and acceleration of necrotic core expansion [4,7]. The clinical importance of this pathway is highlighted by studies showing that elevated IL-1 family cytokines, NLRP3 activation markers, and inflammasome-related macrophage signatures correlate with plaque burden, lesion activity, and cardiovascular events [6,43]. In experimental models, blocking NLRP3 or downstream IL-1 signaling reduces plaque size, limits macrophage infiltration, and improves fibrous cap stability [43,46] (Figure 3). These findings position NLRP3 as a mechanistic link between metabolic stressors, macrophage dysfunction, and chronic arterial inflammation [42,45].

The role of macrophage-mediated inflammation is also supported by the increased expression of TREM1 and TLRs, mainly TLR2 and TLR4, expressed on macrophages, in atherosclerotic plaques, and their association with plaque vulnerability. Activation of TREM-1, TLR-2, and TLR-4 results in activation of cytoplasmic kinases, followed by activation of transcription factors and increased secretion of inflammatory cytokines, which contribute to the formation, progression, and vulnerability of atherosclerotic plaques [47,48,49,50] (Figure 3).

Overall, inflammation is not just a background feature of atherosclerosis. It is the framework through which macrophages regulate plaque biology [4,5]. By driving cytokine production, foam cell dysfunction, and inflammasome activation, macrophages reinforce the inflammatory circuit that promotes plaque vulnerability [2,3,51]. Understanding how pathways like NLRP3 interact with other macrophage-regulating signals, including S100 proteins, is essential for identifying therapeutic targets that can break this cycle and restore immune balance within the arterial wall [21,39].

## 4. S100A8/A9 and S100A12 Proteins in Atherosclerosis and Plaque Vulnerability

S100 proteins are a family of low-molecular-weight, calcium-binding proteins that function as regulators of cellular stress and inflammation [12,52]. Among them, S100A8, S100A9, and S100A12 (hereafter, S100 proteins) have been most strongly linked to cardiovascular disease [14,16]. These proteins are expressed in neutrophils and monocytes and are released during cellular activation or injury, where they act as DAMPs [15,21]. S100A8 and S100A9 are secreted primarily from neutrophils and monocytes, with other immune cells like macrophages and myeloid-derived suppressor cells [53,54]. This release occurs through both active and passive pathways, such as during inflammation or cell death processes like NETosis [54,55]. S100A12 is primarily secreted by granulocytes, particularly neutrophils, and is also secreted by monocytes and keratinocytes, especially when they are activated by inflammatory stimuli or stress [16,56].

Once secreted, S100 proteins bind to pattern recognition receptors (PRRs) such as RAGE and TLR4, amplifying inflammatory signaling pathways such as NF-κB and others that are central to atherosclerosis [15,20]. This triggers a pro-inflammatory and oxidative stress response in cells like endothelial cells, smooth muscle cells, and macrophages, accelerating the progression of atherosclerotic plaques [21,22]. This notion is supported by the findings of increased expression of S100A12 in atherosclerotic plaques [6,17] and the immunopositivity of S100 proteins in the coronary arteries with atherosclerotic lesions [57]. The role of S100 proteins in arterial disease and arterial inflammation is also supported by the elevated levels of S100A8/A9 and S100A12 in patients with active disease among 106 serum samples (50 during active disease) from patients with giant cell arteritis and 32 serum samples (16 during active disease) from patients with Takayasu’s arteritis [58]. The independent association of elevated S100A12 levels with increased major adverse cardiovascular events, along with elevated neutrophil count, further suggests the role of S100 proteins in atherosclerosis-mediated cardiovascular disease [59]. Activation of NF-κB leads to increased production of pro-inflammatory cytokines, production of reactive oxygen species, enhanced immune cell function, such as neutrophil adhesion and migration, and increased cell proliferation and migration, particularly of vascular smooth muscle cells [55,60]. Using HUVEC cells, Zhong et al. reported an important role of S100A8/9 in promoting cell growth and angiogenesis involving RAGE signaling and activation of mTORC2, contributing to intimal hyperplasia [61].

In the context of atherosclerosis, S100A8/A9 and S100A12 promote endothelial dysfunction, monocyte recruitment, and macrophage activation [1,53]. Elevated circulating levels of these proteins have been detected in patients with coronary artery disease, and their expression correlates with plaque burden and disease severity [17,18,62]. Experimental studies further support their pathogenic role: S100A8/A9-deficient mice display reduced lesion formation, while overexpression of these proteins enhances vascular inflammation and lipid accumulation within plaques [53,63]. This evidence suggests that S100 proteins are not merely biomarkers but active contributors to plaque development [19,39].

S100 proteins also directly influence plaque stability. By activating NF-κB and mitogen-activated protein kinase (MAPK) signaling in endothelial cells and macrophages, they drive the secretion of cytokines such as TNF-α and IL-1β and matrix-degrading enzymes like matrix metalloproteinase-9 (MMP-9) [47,53] (Figure 3). These inflammatory mediators weaken the fibrous cap and expand the necrotic core, two hallmarks of vulnerable plaques [3,6]. In addition, S100 proteins enhance oxidative stress and promote the death of foam cells, compounding necrotic core formation and destabilizing the lesion [21,44]. Importantly, S100 protein expression has been associated with acute clinical outcomes; patients with high levels of circulating S100A8/A9 or S100A12 are at an increased risk of myocardial infarction and recurrent cardiovascular events [17,62,64]. Table 1 summarizes the role of S100 proteins in atherosclerosis, inflammatory signaling, and as a biomarker in plaque development, progression, and vulnerability. Collectively, these findings place S100 proteins (S100A8/A9 and S100A12) at the intersection of inflammation and plaque vulnerability [19,39]. They not only recruit and activate immune cells but also perpetuate the inflammatory cycle that drives lesion progression [2,21]. By destabilizing the fibrous cap and enlarging the necrotic core, S100 proteins contribute directly to the transition of stable plaque to unstable plaque [3,6].

Circulating S100 proteins (such as S100A8, S100A9, and S100A12, Table 1) act as both key causative drivers and reflective biomarkers of inflammation in atherosclerosis. S100 proteins are abundantly expressed by myeloid cells (neutrophils and monocytes) and are heavily secreted into the bloodstream during vascular inflammation. High circulating levels strongly correlate with plaque severity and the risk of adverse cardiovascular events. S100 proteins do not just circulate as byproducts; they actively participate in atherogenesis. S100 proteins bind to specific receptors (such as RAGE and TLR4) on the surface of endothelial cells, smooth muscle cells, and leukocytes. This binding triggers positive-feedback signaling cascades (like NF-κB) that amplify inflammation, promote macrophage recruitment, accelerate foam cell formation, and cause plaque instability [14,17,20,21,73].

Given this central role, therapeutic targeting of S100 signaling offers a promising avenue for stabilizing plaques and reducing the risk of adverse cardiovascular events. Komatsu et al. [74] in their study with statin-naïve patients with carotid atherosclerotic plaques reported that a decrease in S100A12 with atorvastatin mirrors the improvement in arterial inflammation, and S100A12 may be a therapeutic target in atherosclerosis. Another evidence of increased S100A9 protein in atherosclerotic plaques comes from increased expression of S100A9 in carotid plaques in a proteomic analysis conducted on 28 carotid plaque specimens from 27 patients [75]. The results of significantly increased S100A12 expression in peripheral arterial disease patients in a study conducted on 105 PAD patients [70] further suggest the pathogenic role of S100 proteins and their utility as biomarkers and therapeutic targets.

## 5. S100 Proteins in Macrophage Polarization

Macrophage polarization plays a central role in the progression of atherosclerosis, a chronic inflammatory cardiovascular disease characterized by lipid accumulation, immune cell infiltration, and plaque remodeling [4,24]. Within atherosclerotic lesions, macrophages adopt a spectrum of functional states ranging from pro-inflammatory, classically activated (M1-like) phenotypes to anti-inflammatory and reparative (M2-like) phenotypes [11,27]. The balance between these states is a critical determinant of plaque growth, necrotic core expansion, and fibrous cap stability [4,9]. In recent years, S100 proteins, particularly S100A8, S100A9, and S100A12, have emerged as important regulators of macrophage polarization within the vascular wall [13,14].

**Figure 4:**
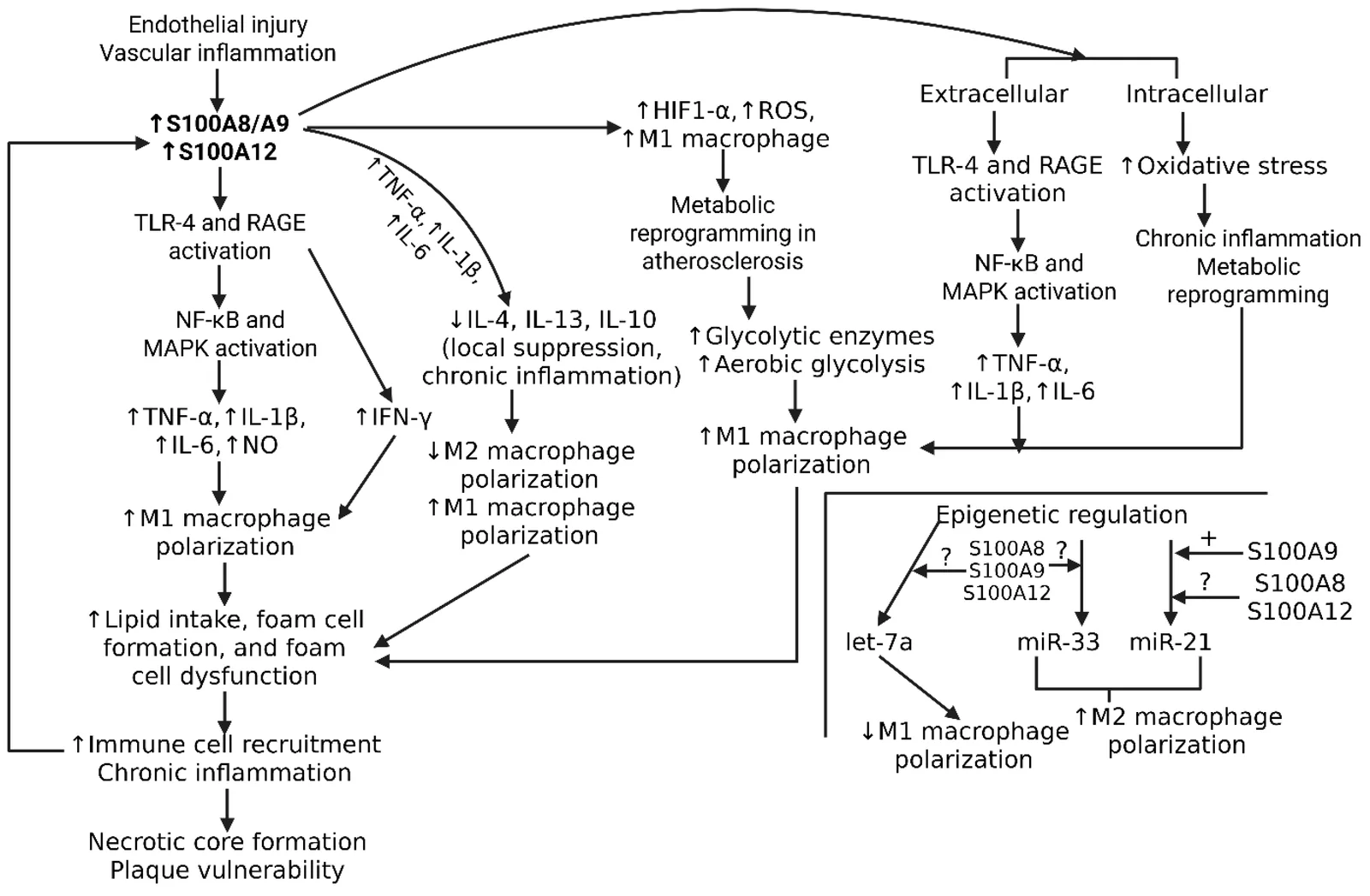
S100A8/A9 and S100A12 in macrophage polarization. Toll-like receptors (TLRs), tumor necrosis factor (TNF)-α, interleukin (IL), nuclear factor kappa beta (NF-κB), receptor for advanced glycation end products (RAGE), mitogen activated phosphokinase (MAPK), nitric oxide (NO), interferon gamma (IFN-γ), reactive oxygen species (ROS). “+” indicate potentiating effect, while “?” indicate unknown effect that needs to be investigated. ↑—increased/activated and ↓—decreased/inactivated. Created in BioRender. Rai, V. (2026) https://BioRender.com/e8znsuz.

In the context of cardiovascular disease, elevated expression of S100A8/A9 has been consistently observed in circulating monocytes, lesional macrophages, and unstable atherosclerotic plaques [23,53]. These proteins act as damage-associated molecular patterns and signal primarily through TLR4 and RAGE, activating downstream pathways such as NF-κB and MAPK that promote inflammatory gene transcription [15,20] (Figure 3). Studies in experimental models of atherosclerosis demonstrate that S100A8/A9 signaling skews macrophages toward a pro-inflammatory phenotype characterized by increased production of TNF-α, IL-1β, IL-6, and inducible nitric oxide synthase [53,76] (Figure 4). This M1-like polarization enhances lipid uptake, promotes foam cell dysfunction, and contributes to defective efferocytosis, all of which accelerate necrotic core formation and plaque vulnerability [4,8]. Activation of TLR-4 and RAGE is associated with the increased secretion of IFN-γ, a mediator that promotes M1 macrophage polarization [77] (Figure 4).

As mentioned above, S100A8/A9 and S100A12 activate TLR-4 and RAGE. This suggests that these S100 proteins, which are increased in atherosclerosis, may contribute to the increased production of IFN-γ and the polarization of M1 macrophages. However, the evidence for this notion is scarcely discussed in the context of atherosclerosis and warrant in depth research. It should also be noted that increased IFN-γ in developing plaque recruits more immune cells, like macrophages and neutrophils [78]. These immune cells may secrete these S100 proteins, making a feed-forward loop for chronic inflammation and other pathologies (Figure 4). Thus, intervening in this loop may be of therapeutic importance. IL-4, IL-13, and IL-10, protective in atherosclerosis, promote M2 macrophage polarization through the Janus Kinases/signal transducer and activator of transcription 3 (JAK/STAT3) and phosphoinositide 3-kinase/protein kinase B/mammalian target of rapamycin (PI3K/Akt/mTOR) pathways, and these mediators suppress S100A8/A9 and S100A12 in macrophages [29,79,80]. Further, increased S100 protein levels in atherosclerosis, in association with decreased IL-4, IL-13, and IL-10 (due to hyperinflammation and local suppression of secretion), will promote M1 macrophage polarization, and attenuating S100 proteins may promote M2 macrophage polarization (Figure 4). Though there is no direct evidence for this, it warrants future research. As discussed above, macrophage polarization involves JAK/STAT3, STAT1, and NF-κB signaling pathways involving various cytokines. These pathways are activated by S100A8/A9 and S100A12 and thus may be of therapeutic importance.

S100A8/A9-mediated macrophage polarization is also closely linked to metabolic reprogramming within atherosclerotic lesions [10,81]. Pro-inflammatory macrophages driven by S100A8/A9 signaling exhibit increased glycolysis and mitochondrial stress, metabolic features associated with sustained inflammatory activation [10,82]. Metabolic reprogramming fundamentally dictates macrophage polarization in atherosclerosis, governing whether these cells drive plaque progression or resolution. Pro-inflammatory M1 macrophages rely heavily on aerobic glycolysis, promoting plaque inflammation and foam cell formation. Driven by the hypoxia-inducible factor 1α (HIF-1α) and signaling pathways like mTORC1, pro-inflammatory macrophages upregulate glycolytic enzymes (e.g., PFKFB3 and PKM2) (Figure 4). This metabolic shift meets the rapid energy demands of an immune response but also traps lipid-laden macrophages in a cycle of inflammation, preventing cholesterol efflux and accelerating cell death (Figure 4). In contrast, anti-inflammatory M2 macrophages utilize oxidative phosphorylation (OXPHOS) and fatty acid oxidation for tissue repair. Anti-inflammatory macrophages rely on mitochondrial respiration and fatty acid oxidation. They promote the resolution of inflammation, clear dead cells through efferocytosis, and aid in tissue repair [10,83,84]. This metabolic shift reinforces inflammatory cytokine production and promotes inflammasome activation, further amplifying local vascular inflammation [43,45]. Increased cholesterol crystals in atherosclerosis are associated with inflammasome activation, contributing to increased secretion of IL-1β and IL-18, which in turn drive the polarization of macrophages into the pro-inflammatory M1 phenotype [42]. S100A8/A9 and S100A12 actively drive metabolic reprogramming in immune and inflammatory conditions. By binding with receptors like RAGE and TLR4, they trigger signaling cascades that shift cellular metabolism away from oxidative phosphorylation toward aerobic glycolysis [33,80], which promotes M1 polarization, as mentioned above (Figure 4). Inhibition or genetic deletion of S100A8/A9 in preclinical cardiovascular models reduces inflammatory macrophage burden and favors a transition toward more reparative macrophage states, supporting a direct role for these proteins in regulating macrophage phenotype during atherogenesis [53,85].

S100A12, another member of the S100 family strongly associated with cardiovascular disease, also contributes to macrophage polarization in atherosclerosis [16,19]. S100A12 is highly expressed in advanced and unstable plaques and signals predominantly through RAGE [17,22]. In macrophages, S100A12 enhances sensitivity to inflammatory stimuli and promotes reactive oxygen species generation, sustaining an M1-like inflammatory environment [16,21] (Figure 4). Importantly, S100A12 has been shown to suppress anti-inflammatory signaling pathways, including IL-10 mediated responses, thereby limiting macrophage transition toward M2-like phenotypes that are associated with tissue repair and plaque stabilization [3,86]. Elevated circulating S100A12 levels correlate with plaque complexity and cardiovascular risk, underscoring its relevance to disease severity [17,59].

While the pro-inflammatory effects of S100 proteins are most evident in atherosclerosis and other cardiovascular diseases, studies from non-cardiovascular contexts provide additional mechanistic insight [13,87]. In models of acute tissue injury and infection, S100A8/A9 can transiently support macrophage-mediated repair and resolution of inflammation [55,88]. However, in chronic inflammatory diseases such as atherosclerosis, diabetes-associated vascular disease, and autoimmune disorders, persistent S100 signaling drives maladaptive macrophage polarization and prolonged inflammation [63,89]. This distinction highlights the importance of disease context and inflammatory duration in determining the functional outcome of S100-mediated macrophage responses [19,39].

Oxidative stress is another mediator that promotes M1 macrophage polarization [90]. S100A8/A9 and S100A12 are key regulators of oxidative stress in the arterial wall. Intracellular S100A8/A9 binds to cytosolic components (such as p47phox and p67phox), enhancing NADPH oxidase activity. This directly amplifies the generation of intracellular ROS [21]. Extracellular S100A8/A9 and S100A12 interact with endothelial cells, macrophages, and vascular smooth muscle cells primarily via RAGE and TLR4. These binding triggers downstream pathways—such as NF-κB and MAPK—resulting in continuous ROS production and a pro-inflammatory feedback loop [14,62,91] (Figure 4). These findings suggest that targeting S100A8/A9 and S100A12-mediated vascular inflammation involving ROS and macrophage polarization may be of therapeutic importance, but this concept warrants research.

Epigenetic regulation of macrophage polarization is another aspect that should be focused on for the therapeutic aspects of atherosclerosis. MicroRNAs play a direct role; for instance, miR-33 and miR-21 promote M2 polarization, while let-7a can inhibit M1 polarization in autoimmune diseases [92] (Figure 4). MicroRNA let-7a acts as a double-edged sword in atherosclerosis. While generally protective by suppressing vascular inflammation and cell proliferation, its sequestration by the long non-coding RNA H19 (in the H19/let-7a/ITGB3 axis) unleashes inflammatory pathways that actively drive endothelial dysfunction and plaque progression [93]. miR-33) is a key regulator of cholesterol homeostasis and lipid metabolism. Encoded within the SREBF genes, miR-33 represses genes like ABCA1 and downregulates autophagy pathways. miR-33 represses ABCA1 and ABCG1, which are critical transporters that move excess cholesterol from macrophages to HDL. This blockade causes macrophages to accumulate lipid droplets, transforming them into foam cells that form atherosclerotic plaques. miR-33 targets and represses critical autophagy regulators (such as FOXO3 and TFEB) in macrophages. By blocking autophagy, miR-33 restricts the breakdown of lipid droplets and the digestion of apoptotic cells, thereby driving chronic inflammation and hindering plaque clearance. Therapeutic silencing of miR-33 using Anti-miR-33 Oligonucleotides promotes plaque regression, boosts high-density lipoprotein (HDL) function, and reprograms the immune landscape of lesions [94,95,96]. Using a mouse model, the role of miR-33 in regulating the immune cell landscape in atherosclerotic plaque was investigated, and the study using anti–miR-33 treatment of *Ldlr*^−/−^ mice reported increased macrophage attrition by apoptosis and efferocytotic clearance, decreased circulating monocytes and splenic myeloid progenitors, decreased macrophage proliferation and retention by inhibiting miR-33 [97]. These findings suggest that miR-33 plays a regulatory role in atherosclerosis. In metabolic conditions (like atherosclerosis or metabolic syndrome), miR-33 expression influences inflammatory cytokine secretion and macrophage behavior, linking lipid pathways with S100-mediated inflammation [98]. miR-21 is a critical, cell-specific regulator of atherosclerosis that presents a therapeutic paradox. The role of miR-21 in plaque development is highly dependent on both the stage of the disease and the specific cell type involved. In macrophages, it acts as an anti-inflammatory agent that prevents foam cell formation and stabilizes plaques. However, its overexpression in vascular smooth muscle cells drives lesion growth and restenosis. In the inner lining of the blood vessels, miR-21 stimulates the synthesis of nitric oxide (NO), which helps relax blood vessels, but it can simultaneously promote localized inflammation [99,100,101]. In many chronic inflammatory conditions and late-stage sepsis, S100A9 acts as an intracellular transcription cofactor. It associates with and stabilizes the Stat3-C/EBPβ protein complex, which directly binds to the promoters of the miR-21 gene to induce its expression [102]. However, the association of S100A8/A9 and S100A12 with miR-21 in the context of atherosclerosis is minimally explored and warrant research. There is no direct evidence of the regulation of let-7a and miR-33, but inflammatory signaling mediated by S100A8/A9 and S100A12 may affect their expression and warrant investigation (Figure 4).

Taken together, current evidence supports a model in which S100A8, S100A9, and S100A12 act as amplifiers of pro-inflammatory macrophage polarization in atherosclerosis, reinforcing chronic vascular inflammation and contributing to plaque instability [13,21]. By promoting inflammatory signaling, metabolic dysfunction, and impaired reparative responses in macrophages, these proteins sustain the pathogenic immune environment characteristic of advanced cardiovascular disease [10,81]. These findings position S100 proteins not only as biomarkers of atherosclerotic inflammation but also as active regulators of macrophage phenotype and promising targets for therapeutic intervention aimed at restoring immune balance within atherosclerotic plaques.

## 6. S100 Protein Targeting on Macrophage Polarization in Atherosclerosis

Given the central role of S100 proteins in driving inflammatory macrophage polarization and plaque instability, targeting S100-mediated signaling has emerged as a promising therapeutic strategy in atherosclerosis [19,39] as well as thrombosis [103]. By modulating macrophage phenotype, these approaches aim to reduce chronic vascular inflammation, enhance plaque stability, and ultimately lower the risk of cardiovascular events [4,5]. Targeting macrophages and mediators associated with macrophages has been shown to play a critical role in plaque formation and vulnerability and targeting them as potential therapeutics. For instance, triggering receptor expressed on myeloid cells (TREM)-1 is associated with plaque vulnerability [47,48,104], and targeting TREM-1 limits the development of atherosclerotic plaque [105]. Several therapeutic strategies have been explored, including direct inhibition of S100 proteins, blockade of their receptors, and downstream suppression of inflammatory signaling pathways [20,21].

One approach involves directly targeting S100A8 and S100A9, which are major drivers of pro-inflammatory macrophage activation [53,63]. In experimental models, genetic deletion or pharmacological inhibition of S100A8/A9 reduces macrophage infiltration, inflammatory cytokine production, and plaque burden [63,85]. These interventions also promote a shift toward more reparative macrophage phenotypes, improving efferocytosis and limiting necrotic core expansion [7,8]. Importantly, inhibition of S100A8/A9 does not completely suppress immune function, suggesting that selective modulation of inflammatory signaling may be achievable without broad immunosuppression [39,88].

Another therapeutic strategy focuses on blocking receptors through which S100 proteins exert their effects, particularly RAGE and TLR4 [15,45]. RAGE inhibition has been shown to reduce NF-κB activation, oxidative stress, and inflammatory cytokine production in macrophages and vascular cells [16,22]. Similarly, TLR4 blockade attenuates S100A8/A9-induced macrophage activation and limits inflammasome signaling within plaques [15,43]. These receptor-targeted approaches disrupt the amplification loop between S100 proteins and inflammatory macrophages, thereby dampening chronic vascular inflammation [2,21].Targeting downstream inflammatory pathways activated by S100 signaling also offers therapeutic potential. S100-induced activation of the NF-κB and MAPK pathways promotes expression of cytokines such as IL-1β, TNF-α, and IL-6, as well as matrix-degrading enzymes that destabilize plaques [3,47]. Pharmacological inhibition of these pathways has been shown to reduce macrophage-driven inflammation and improve plaque stability in preclinical studies [43,46]. Additionally, suppressing NLRP3 inflammasome activation downstream of S100 signaling decreases IL-1β production, limits pyroptosis, and reduces necrotic core formation [41,44].

Despite promising preclinical data, translating S100-targeted therapies into clinical practice presents several challenges. S100 proteins play context-dependent roles in immune defense and tissue repair, raising concerns that systemic inhibition could impair host responses or delay resolution of acute inflammation [87,88]. Furthermore, redundancy within inflammatory signaling networks may limit the efficacy of targeting a single molecule or pathway [21,45]. These challenges highlight the importance of developing strategies that selectively target pathogenic S100 signaling within atherosclerotic plaques while preserving protective immune functions [19,39].

In summary, S100 proteins regulate macrophage-mediated inflammation [13], which in turn plays a critical role in the pathogenesis of atherosclerosis and plaque vulnerability. A dynamic balance between M1 and M2 macrophages is a must to regulate various factors involved in the progression of atherosclerotic plaques [4,47]. Since M1 macrophages in the plaque enhance plaque vulnerability, while M2 macrophages are associated with decreased inflammation and regression of atherosclerosis [106,107], targeting macrophage polarization towards the M2 phenotype may be beneficial to attenuate chronic inflammation and plaque progression toward vulnerability. Activation of TLR-4 results in increased expression of PLD1, the instigator for M1 macrophage polarization, while PLD2 polarizes macrophages towards the M2 phenotype. Inhibition of PLD1 results in decreased M1 macrophages and increased M2 macrophages [108]. It is important to note that S100A8, S100A9, and S100A12 are associated with the activation of TLR2, TLR-4, and RAGE, and inhibiting these protein-mediated TLR-4 and RAGE activation may be a promising approach to attenuating atherosclerosis [14,16,21]. Since activated TLR-4 is involved in PLD1 activation, targeting S100A8, S100A9, and S100A12 to downregulate TLR-4, thereby PLD-1 and M1 macrophages may be a promising approach to attenuate plaque inflammation and vulnerability.

Collectively, this suggests that inhibiting the S100 protein axis may attenuate inflammation by increasing M2 macrophage polarization, resulting in decreased secretion of inflammatory cytokines and metalloproteinases, and favorable collagen remodeling leading to attenuation of plaque vulnerability and progression. However, it should be noted that there are currently no clinically approved plaque-specific therapies specifically targeting the S100 protein family (S100A8/A9, S100A12) for atherosclerosis. Continued research into the precise mechanisms of S100 signaling and macrophage regulation will be critical for designing strategies toward safe and effective clinical applications. However, it should be noted that designing and translating S100-tragted therapies in atherosclerosis is not easy because of complex receptor redundancies (TLR-4 and RAGE), dual pro- and anti-inflammatory roles, and the difficulty of delivering therapeutics to specific plaque microenvironments without triggering systemic immune suppression. Blocking a single S100 protein or receptor often leads to compensatory signaling pathways, reducing overall therapeutic efficacy. Atherosclerotic plaques contain a complex mix of cell types, including endothelial cells, macrophages, and smooth muscle cells, which process S100 signals differently. A therapy that works on one cell type may have unintended pro-inflammatory effects on another. Systemic administration of S100 inhibitors could lead to unwanted broad immunosuppression or toxicity (off-target effects). Another issue is of translational gap in animal models. Preclinical models typically fail to fully replicate human plaque rupture and the complex, prolonged progression of the disease in older patients, making it difficult to predict clinical outcomes [21,73,109].

## 7. Future Directions

S100 proteins have emerged as key mediators of macrophage polarization and drivers of chronic vascular inflammation in atherosclerosis [12,13]. Acting through RAGE and TLR4, they shift macrophages toward pro-inflammatory states, impair efferocytosis, and contribute to necrotic core expansion, all of which promote plaque vulnerability [7,15,16]. In addition, their role as damage-associated molecular patterns positions them not only as pathogenic agents but also as biomarkers of disease activity and progression [17,19]. Elevated circulating levels of S100A8/A9, for example, have been correlated with adverse cardiovascular outcomes, further underscoring their clinical significance [21,63].

Therapeutically, S100 proteins present both opportunities and challenges. Their inhibition has been shown to reduce inflammatory signaling, improve macrophage balance, and stabilize plaques in preclinical studies [20,39]. Yet their essential roles in host defense and tissue repair highlight the risks of indiscriminate blockade [87,88]. The future of this field lies in strategies that carefully balance suppression of pathological S100 activity with preservation of their physiological functions [21,45]. This might involve precision approaches such as nanoparticle-based drug delivery directly to inflamed vascular sites, transient inhibition during acute inflammatory flares, or the development of drugs that selectively modulate downstream signaling without eliminating S100 function [5,39].

Ultimately, atherosclerosis remains a disease of unresolved inflammation, and S100 proteins sit at the crossroads of immune activation and macrophage behavior [2,4]. By deepening our understanding of how S100 proteins (S100A8/A9 and S100A12) regulate macrophage polarization and plaque biology, researchers can design targeted therapies that not only slow disease progression but also reduce the risk of plaque rupture and cardiovascular events [19,20]. Such advances hold the potential to translate into significant clinical benefits, making S100 proteins a compelling focus of future cardiovascular research [19,39]. Use of multi-omics (transcriptomics, proteomics, and epigenomics) to reveal factors regulating macrophage polarization within the plaque beyond binary state and identifying highly localized, dynamic subsets that alter lipid handling and plaque stability. These omics may also help in identifying novel checkpoints that trigger redox imbalances and ferroptosis in macrophages. Spatial omics has redefined atherosclerosis research by mapping how specific macrophage polarization states assemble into distinct cellular neighborhoods within the vascular wall. This technology moves beyond the simplified M1/M2 binary, revealing specialized, location-dependent macrophage subsets driving plaque progression and instability [110,111]. Translating preclinical study results to clinics faces hurdles, as discussed above, and to reduce off-target effects, targeted delivery of macrophage-targeted therapy may be helpful. Macrophage-targeted drug delivery in atherosclerosis utilizes engineered nanomedicines to direct anti-inflammatory, lipid-regulating, and plaque-stabilizing therapeutics straight to lesions. This approach limits off-target toxicities and enhances efficacy by exploiting overexpressed macrophage receptors (e.g., scavenger and mannose receptors) or utilizing biomimetic cell-membrane coatings [112,113]. Precision immunomodulation targeting specific inflammatory pathways rather than broadly suppressing the immune system may be another approach to target macrophage polarization in atherosclerosis. This may be achieved using biomimetic platforms like nanoparticles and spatiotemporal release of drugs where vehicles are designed to trigger drug release only upon encountering disease-specific microenvironments [114].

## 8. Conclusions

Atherosclerosis remains a disease of unresolved inflammation, and S100 proteins sit at the crossroads of immune activation and macrophage behavior. Deepening our understanding of how S100 proteins (S100A8/A9 and S100A12) regulate macrophage polarization and plaque biology, targeted therapies that not only slow disease progression but also reduce the risk of plaque rupture and cardiovascular events may be designed. Focus should be on developing strategies on the translational aspects of preclinical studies to clinics.

## Figures and Tables

**Table 1:** S100 proteins as biomarkers in atherosclerosis and cardiovascular events. Most of these studies were observational studies, and the cause-and-effect relationship was not investigated.

S100 Protein	Patient Population	Study Outcome
S100A12 [17]	Plasma levels of S100A8/A9 and S100A12 in 159 patients with high-grade carotid stenosis and in 22 healthy control subjects.	Significantly increased plasma levels of S100A12 in patients with carotid atherosclerosis. Highest S100A12 levels were associated with recent (≤2 months) symptomatic plaque. Plasma levels of S100A8/S100A9 showed a modest increase in patients with symptoms between 2 and 6 months but not in other patients. Increased gene expression of S100A8, S100A9, and S100A12 in symptomatic patients. S100A12 may be related to plaque instability. S100A8/A9 are atherogenic and S100A12 promote plaque vulnerability.
S100A8/A9 [65]	60 coronary artery disease (CAD) patients with psychological stress and 60 controls	S100A8/A9 correlate with poor prognosis in CAD Dysregulated cortisol secretion in CAD patients may be associated with an exaggerated pro-inflammatory S100A8/A9 response. S100A8/A9 are both cause and effects- increased stress increase S100A8/A9 secretion and their increase promote inflammation.
S100A8/A9 [62]	1062 patients with acute myocardial infarction (MI) and 1043 validation cohorts.	S100A8/A9 is a predictor and potentially causal mediator for heart failureafter an acute MI. Predictive value of S100A8/A9 is superior to that of the traditional biomarkers. The addition of S100A8/A9 improves the risk estimation using traditional risk factors. S100A8/A9 has causal-effects for heart failure.
S100A12 [66]	2443 patients with drug-eluting stent-based percutaneous coronary intervention (258 patients with in-stent restenosis and 258 patients without stenosis).	Increased serum levels of S100A12 are associated with in-stent restenosis after drug-eluting stent placement. S100A12 may be an independent marker to predict restenosis- suggesting is causal effect on restenosis.
S100A12 [67]	90 patients with coronary angiography and/or percutaneous coronary interventions	Increased serum levels of S100A9 in ACS patients compared to normal and of S100A12 in ACS compared to normal and stable coronary arteries. S100A12 may serve as a marker of coronary plaque instability in acute coronary syndrome (ACS). S100A12 may serve as an independent marker to predict in-stent restenosis.
S100A8/A9 [68]	39 patients with stable angina and 53 patients with unstable angina	S100A8/A9 complex is involved in coronary atherosclerotic plaques inflammation in patients with unstable angina. A causal effect of S100A8/A9 in inducing inflammation in plaque rendering it unstable may be possible.
S100A12 [69]	1023 patients with acute angina.	S100A12 may be an early biomarker in patients with ST-segment elevation myocardial infarction (STEMI).
S100A12 [70]	105 patients with peripheral arterial disease (PAD) and 373 controls.	Significantly higher levels of S100A12 is associated with PAD. Elevated S100A12 level is an independent risk factor for PAD in patients with dyslipidemia.
S100A12 [71]	652 patients with stable coronary artery disease (CAD).	S100A12 could be a novel biomarker for predicting cardiovascular events in patients with CAD.
S100A12 [72]	1345 type 2 diabetes patients	Increased serum S100A12 levels are independently associated with risk of heart failure but not with risk of major adverse cardiovascular events.

## Data Availability

No new data were created or analyzed in this study. Data sharing is not applicable to this article.
